# Determinants of modern contraceptive use among married Somali women living in Kampala; a cross sectional survey

**DOI:** 10.1186/s12978-020-00922-x

**Published:** 2020-05-24

**Authors:** Maryan Abdulahi, Othman Kakaire, Fatuma Namusoke

**Affiliations:** Department of Obstetrics and Gynaecology, School of Medicine, Makerere University , 7062, Kampala, Uganda

**Keywords:** Modern contraceptives use, Somali women, Kampala

## Abstract

**Background:**

Low contraceptive uptake exposes women to unintended pregnancies and often the resultant obstetric complications. The immigrant communities especially from countries with low contraceptive use present a unique challenge. The main objective of the study was to describe modern contraceptive use and associated factors among married Somali women living in Kampala.

**Methods:**

A community based cross sectional survey was conducted among Somali women living in Kampala from August to November 2016. Using consecutive sampling, 341 respondents were recruited after informed consent. Data was collected using interviewer administered questionnaires on contraceptives use and factors associated. Data was entered in Epidata 3.1 and analyzed using STATA 11.0. Logistic regression analysis was used to determine the factors associated with use of modern contraceptives.

**Results:**

Majority of the participants were refugees 317/341(94%), with a mean age of 30.4 (±6.7) years and 136/341 (40%) had no formal education. More than 325/341 (95%) of respondents desired to have five or more children (Average 9 + 3) and 164/341 (45%) had five or more living children. Only 29% of women were using modern contraceptives, of which 51% used oral pills, 15% condoms and 15% injectables. Having tertiary education, one who had ever used modern contraceptives and desire for spacing of more than two years were independently associated with current of use modern contraceptives.

**Conclusions:**

The Contraceptive prevalence rate among married Somali women in Kampala was (29%). A majority of the respondents were using short acting contraceptive methods. Attaining tertiary education, ever use of modern contraceptives, those whose source of contraceptive information was health worker and desire to space for more than two years were associated with current use of modern contraceptives. There is a need for improvement of girl child education, contraceptive awareness and male involvement to increase contraceptive uptake in this community. Research looking at attitude of this community towards use of long term contraception is recommended.

## Plain English summary

Low utilization of modern contraceptives among married couples leads to unwanted pregnancy which is associated with an increase in maternal morbidity and mortality. The situation of the immigrants in regards to modern contraceptive use is worse although the extent is not adequately documented. The aim of the study was to assess the prevalence and factors associated with modern contraceptives use among married Somali women living in Kampala. Three hundred forty one participants were recruited in a community based, cross sectional survey in Rubaga Division, Kampala. The contraceptive prevalence was 29% with majority (70%) using short term contraceptives. The women who were more educated, one who had ever used modern contraceptives, got contraceptive information from the health worker and/or desired spacing of more than two years were more likely to use modern contraceptives. There is need to increase girl child education and male participation in these communities to improve utilization of modern contraceptives. We recommend studies looking at attitudes of this population towards use of long term contraceptives.

## Introduction

Access to family planning (FP) is critical to women and children’s health. Globally 303,000 women were estimated to have died from pregnancy and childbirth related complications in 2015 [1]. There is huge gap between the need for and access to contraceptive services leading to the high unmet need for contraceptives [2].

The situation on the unmet need for contraception is even dire among people of Somali decent. Somalia has been undergoing through conflict for more than two decades since the central government collapsed in 1991. This has led to displacement of people internally as well as immigration with an estimated 810,287 refugees living in different countries [3] . Conflict areas tend to have higher under-five and maternal mortality [4] and high unmet need for contraceptives [5]. This has been attributed to the disproportionate distribution of funds with more emphasis being put on defense and disruption of the health care delivery systems.

Somalia a predominantly Muslim country has one of the highest fertility rates of 6.6 children per woman of reproductive age and modern contraceptive prevalence rate of 1% [6, 7]. There is generally a high value of having children in this community and husband approval for the women to use contraceptives is very critical in this population [8].

In refugee settings the factors that are associated with use of contraceptives include access to service delivery points, inadequate knowledge on the different methods, fear of side effects and religious and cultural barriers with adolescent unmarried women most affected [5]. Data on the knowledge, fertility desires and current use of modern contraceptives in married Somali women living in Kampala is lacking. The aim of the study was to determine the prevalence and factors associated with use of modern contraceptive among married Somali women living in Kampala.

## Methods

### Study design

This was community based cross-sectional study done from August to November 2016.

### Study site

The study was carried out in Rubaga division one of the seven Divisions which make up Kampala Capital City. Rubaga division was purposively selected since according to Kampala Capital City Authority (KCCA), majority of Somali people live in Rubaga division. According to UNHCR Registered Somali Refugee Population in Uganda in 2015 were estimated to be 41,014 comprising of 48% females and 52% males. By September 2016, about 15,573 of them were in Kampala while 22,474 were staying in Nakivali refugee camp in western Uganda reference [3]. There were no registered households where the Somali live because most of live in informal settlements around Kampala City.

Rubaga Division is located in the western part of the city bordered by Wakiso district to the west and south, Kampala Central Division to the east, and Kawempe Division to the northern part. Administratively, the division is made of 13 parishes – in which there are an equal number of informal settlements. The informal settlements in this part of Kampala are; Busega, Kasubi, Kawaala, Kizito Block Najja II, Kosovo (Bukooza), Lungujja-Kintunzi, Mutundwe-Wabiyinja, Najjanankumbi, Namirembe Bakuli, Namungoona, Nankulabye, Nateete, Ndeeba and Wankulukuku. The geographical extent of Rubaga division occupies a total of 9110 acres of land. Of this, the slum settlements occupy only 1981 acres which accounts for 22% of the whole division. There are approximately 414,750 people in the informal settlements of Rubaga division.

### Study population

The inclusion criterion was a married Somali woman of reproductive age (18–49 years) who had lived in Uganda for less than 10 years. The potential participants were excluded when they were too ill to answer the questions.

### Data collection

Study participants were approached, screened and recruited into the study after informed written consent. Consecutive sampling of households of Somali families was done. There are no organised settlements in the study areas. The households were identified by the research assistants with the help of study participants. Two research assistants who stay in Rubaga Division of Somali origin with the help of some research participants were able to identify the households of interest. Two educative sessions about cervical cancer screening and exclusive breastfeeding were initially held in one of the houses for Somali women who stay in Rubaga by one of the investigators and research assistants, which helped us access more participants.

Quantitative data was obtained using a structured interviewer administered questionnaire. The questionnaire was developed using validated questions from Demographic Health Surveys, background characteristics, reproductive behavior and intentions and contraception [9]. Details on current use of modern contraceptives the type of contraception which was used were collected. The independent variables included age of the woman, religion, level of education of the woman and partner, marital status, occupation. Reproductive history and fertility desire data was collected which included; parity, number of living children, desire for more children, desired future child spacing, contraceptive awareness, source of information about contraceptives, ever use of contraceptives and partner’s approval for contraceptive use.

### Main outcome variable

Modern contraceptive use among currently married Somali non-pregnant women of reproductive ages 18–49 years was collected. Women within the ages 18–49 years were asked if they used any contraceptive to delay or avoid conception. Modern contraceptives methods included pills, female and male sterilization, Intrauterine Device (IUD), injectable, implants, male and female condom, diaphragm, emergency contraception and lactation Amenorrhea Method (LAM).

### Quality control

The data was collected with help of research assistants who were fluent in both English and Somali languages. These were trained for two days by the team on the data collection methods, protocol and consenting process. The research assistants were female with a medical background (Nurse and clinical officer).

The questionnaires were pre-tested on 10 women in Kisenyi before adoption for data collection with a purpose of checking clarity, suitability of questions, identification of repetitions, irrelevance and inconsistence. After pretesting, the questionnaire was revised and further refined during training prior to actual data collection.

### Sample size calculations

The sample size was calculated by adopting the proportion of 26% which is the modern contraceptive prevalence rate in Uganda (UDHS 2011). A maximum likely error of 5% with a 95% confidence interval was used. An allowance of 10% was estimated for the possible non-response. The sample size was calculated using the Kish Leslie formula (1965) for cross sectional studies which gave a total 341 participants. A total of 381 participants were approached and 40 declined to take part or complete the interview.

### Statistical analysis

Data was entered in Epidata version 3.1 software and exported to STATA 11.0 for analysis. Descriptive analysis was done by calculating mean and standard deviation for continuous and proportion for categorical variables. To identify factors associated with modern contraceptive use, bivariate logistic regression between outcome variable (modern contraceptive use & non-use) and independent variables (age, education of respondent, education of the husband, employment status, exposure to family planning messages, husband approval) was carried out. Crude odds ratio (OR) and their 95% confidence intervals (CI) were calculated. Variables with *p*-value < 0.02 or which are biologically important were selected for multivariable logistic regression analysis. The final multivariable logistic regressions was performed to assess the independent effects of individual factors by controlling potential confounders and adjusted OR (AOR) with their 95% CI was computed using a backward elimination method.

## Results

The majority of the enrolled participants were refugees 317/341 (93%), of which 133/317 (39%) had lived in Uganda for less than one year. Majority 249/341 (73%) were housewives and 136/341 (40%) did not have any formal education. The details of the demographics characteristics are shown in Table 1.

**Table 1:** Socio Demographic characteristics of the Study participants

Variables	Frequency(*n* = 341)	Percentage (%)
**Age of the participant**		
Less than 25 years	66	19
25–29 years	103	30
30–34 years	89	26
35–39 years	38	11
> =40 years	45	13
Residence status		
Refugee	317	93
Resident	24	7
Length of stay in Uganda		
less than 1 year	133	39
1 to 5 years	160	47
6-<10 yrs	48	14
Women’s education status		
Informal/none	136	40
Primary	49	14
Secondary	94	28
Tertiary	62	18
Employment status		
Housewife	249	73
Informal employment	48	14
Student	29	9
Professional/others	15	4
Husband education status		
Informal	137	40
Primary	10	3
Secondary	72	21
Tertiary	122	36

### Reproductive history and fertility intentions of the study participants

Almost half of participants 158/341 (46%) were less than 20 years at marriage and 164/305 (45%) had five or more living children. Nearly all participants 325/341 (94%) desired to have more than five children (average of 9) but with a desire for spacing of two or more years 208/305 (69%). Other details of fertility intentions are shown in Table 2.

**Table 2:** Reproductive history and fertility intentions of study participants

Variables	Frequency(*n* = 341)	Percentage (%)
Age at marriage		
Less than 20 years	158	46
20 to 25 years	157	46
26 years and more	26	7
Have you ever got pregnant		
Yes	305	89
No	36	11
Parity		
0	36	11
Less than 4	123	36
4 or more	182	53
^a^Current living children	**305**	
1 child	30	11
2–4 children	111	44
> =5 children	164	45
Space between children	**286**	
Less than 2 years	157	55
2 years	103	35
More than 2 years	26	10
Desired number of children by woman	**340**	
< 5	15	4
> =5	325	96
Desired number of children by husband	341	
Same number	62	18
More children	120	35
Less children/Don’t Know	159	47
^a^Desired future spacing	305	
< 2 years	97	31
2 years	160	53
> 2 years	48	16

^a^Only women with children included

### Awareness and ever use of modern contraceptives

The majority of participants 273/314 (80%) had ever heard about modern contraceptives.

More than half of them 138/273 (51%) had heard about modern contraceptives from a health worker, while 73/273 (27%) from a friend. Among women who had ever heard about modern contraceptives, 192/273 (70%) had ever used at least one of the modern contraceptives. Short acting contraceptives like oral contraceptive pills 83/192 (43%) and condoms 58/192 (30%) were the most commonly ever used contraceptives. More than half 150/341 (55%) of the women had ever discussed use of modern contraceptives with their husbands but only 124/273 (46%) of them got their husbands approval to start use of modern contraceptives yet for 255/273 (94%) of the women felt that they can only proceed to use after the approval. Details on awareness and ever use of modern contraceptives are shown in Table 3.

**Table 3:** Awareness and ever use of modern contraceptives

Variables	Frequency(*n* = 341)	Percentage (%)
Women who are aware about modern contraceptives		
Yes	273	80
No	68	20
Source of information about contraceptives		
Heath worker	138	51
Media	13	5
Newspaper	4	1
Television	23	8
Radio	10	4
Internet	23	8
Friend	73	27
Family member	14	5
Believes husband’s approval is a must before use	255	94
Had ever discussed contraceptives with their husbands	150	55
Got husband approval	124	46
Ever used modern contraceptives		
Yes	192	56
No	149	44
Methods used *192		
condom	58	30
Oral pills	83	43
Injectables	26	14
implants	12	6
Intrauterine Device	6	3
Vaginal rings	7	4

### Prevalence of modern contraceptive use among married Somali women in Kampala

The study found that 100/341 (29%) of married Somali women living in Kampala were using modern contraceptives at 95% confidence interval (24.6–34.6). More than half of these were short term contraceptives; 51/100 (51%) were using oral pills and 15/100 (15%) condoms. Eight participants declined to respond to the reason why they chose the method they were using, lack of side effects 35/92 (38%) and availability 14/92 (15%) were the main reasons for choosing that method. Details on the current methods used by the study participants are shown in Table 4.

**Table 4:** Current use of modern contraceptives among married Somali women in Kampala

Variables	Frequency(*n* = 100)	Percentage (%)
Contraceptive method used currently		
Condom	15	15
Oral pills	51	51
Injectables	15	15
Implants	7	7
Intrauterine Device	4	4
Vaginal rings	4	4
Combination of methods	2	2
Others	2	2
^a^Reasons for choosing current methods		
Available	14	15
User controlled	12	13
No side effects	35	38
Doctor's advice	10	11
Cheap	8	9
Combination	2	2
Others	11	12
Side effects		
Bleeding	19	19
Pain	36	36
Abnormal blood clot	2	2
Delay fertility return	3	3
Unwanted weight gain	10	10
Others	19	19

^a^8 people didn’t respond

### Actors associated with current use of modern contraceptives among Somali women living in Kampala

Majority 98/100 (98%) of the participants who were using modern contraceptives had ever used previously. The factor of ever use of modern contraceptives was not included in the multivariate model. After adjusting for other factors, the following factors were found to be associated with current use of modern contraceptives; the participants who had attained tertiary education had four times higher odds of using modern contraceptives. The participants who desired spacing for more than two years, those whose husbands desired more children than them and where the source of information on modern contraceptives was health worker were more likely to use modern contraceptives after multivariate analysis. Details on the factors that influenced current use of modern contraceptives after multivariate analysis are shown in Table 5.

**Table 5:** Factors associated with current use of modern contraceptive after multivariate analysis

Variables	Adjusted OR	95% Confidence Interval	*P*-value
Age of the participant			
Less than 20 years	1		
20–24 years	0.895	0.2–4.2	0.888
25–29 years	1.594	0.5–4.9	0.417
30–34 years	0.888	0.3–2.5	0.823
35–39 years	1.143	0.3–3.8	0.827
> =40 years	1		
Level of education			
Informal	1		
Primary	0.867	0.3–2.4	0.781
Secondary	1.167	0.5–2.6	0.706
Tertiary	**4.342**	1.7–10.8	0.002*
Parity			
0	1		
1	1.995	0.2–18.9	0.548
2	1.934	0.2–18.0	0.562
3 or more	1		
Space between children			
Less than 2 years	1		
2 years	1.201	0.6–2.4	0.608
More than 2 years	**3.240**	1.0–10.1	0.043*
Desirable number of children by husband			
Same number as woman	1		
More children than woman	**2.748**	1.1–6.9	0.032*
Less children/Don’t Know	2.143	0.8–5.7	0.129
Source of information about contraceptives			
Heath worker			
Yes	**3.003**	1.6–5.8	0.001*
No	1		
Desired future spacing			
< 2 years	1		
2 years	2.045	0.9–5.0	0.075
> 2 years	**3.511**	1.3–9.8	0.016*

*P value less the 0.005

## Discussion

This study looked at the factors associated with modern contraceptive use among married Somali women living in Kampala. Previous use of modern contraceptives, having attained tertiary education, desire for child spacing of more than two years, husband desire for more children and those who got the information of modern contraceptives from the health worker were more likely to use modern contraceptives. The prevalence of modern contraceptive use among married Somali women living in Kampala was found to be 29% that is higher than prevalence of modern contraceptive use in Somalia which is 1% [7], but lower than the current prevalence of the country (Uganda) which is 39% [10]. Studies have demonstrated that immigrant Married Somali women generally have higher modern contraceptive rates than their counterparts living in Somalia. Living in a new community may greatly improve the decision making and discussion about contraceptives amongst the couples [11] and yet in some cases the general practitioners are less likely to discuss contraceptive with the refugee and migrant women especially of African descent than the natives [12]. Studies have demonstrated differences in modern contraceptive prevalence in immigrant population, with some showing a lower prevalence than resident women [13] and others having a comparable contraceptive prevalence rates [11, 14]. The differences can be explained by the different populations with those with a very high prevalence in hospital based studies and there are differences in the definition of contraceptive prevalence with some studies including traditional contraceptive methods and others not.

A significant proportion (40%) of the participants did not have any formal education or had attained only primary education (14%). The participants who had attained tertiary education were four times higher odds of using modern contraceptives than those who had no formal education after controlling for confounders. This finding is consistent with other studies showing that educated women were more likely to use modern contraceptives than their less educated counterparts [15–17]. Formal education is likely to influence the attitudes, knowledge and access to health services. Knowledge and access are very important in determining those who will use the modern contraceptives.

Nearly three quarters of the women desired child spacing of two or more years though only 29% were currently using, the remaining 39% may have had unmet need for contraception. The women who desired spacing of more than two years had higher odds of using modern contraceptives. A study done in Uganda showed that women with future desire for children, would like to have two or more years of spacing [18]. Potential contributing factors to the high unmet need for contraceptives include failure to get husband’s approval, lack of awareness about modern contraceptives, or fear of side effects. A systematic review of studies done in SubSaharan Africa, male partner approval was found to negatively affect contraceptive use [19]. Another possible explanation can be absence of husband, where the couple may not stay together sometimes because of refugee status. Majority of participants had ever heard about modern contraceptives and yet only half of these got the information from a health worker. Participants who had got the information of modern contraceptive form a health worker were independently more likely to use modern contraceptives. In a study where majority of the participants got information from television only attitude of the participants and religious influence affected use of modern contraceptives [20]. Information from a health worker is likely to be accurate and gives an opportunity to address any misunderstanding. Participants who get the information from friends on other hand are likely to get distorted and incomplete information that hinders them from using modern contraceptives.

Women who had ever used any modern contraceptive were more likely to currently use contraceptives in this population. The main reason for choosing the contraceptives was the lack of side effects. It’s likely ever use of contraceptives dispelled all the different myths and these had realised the benefits of contraceptive use.

### Strengths and limitations

The main strength of this study was that, being community based, it could reflect the actual experience of the married Somali women during the study period. This looks at a special group of refugees not living in camps. It’s a big sample size which can be generalizable to this special group. One limitation of this study was the use of consecutive sampling, as well as snow ball recruiting to identify potential participants. This may affect the representativeness of the interviewed population, although the large number of participants should help to limit his concern. This was done to enable us reach as many of the potential participants who normally stay at home. This is a true representation of this community since these tend to live in the same neighbourhood.

## Conclusion

In this largely refugee married Somali population living in Kampala, the modern contraceptive prevalence among married women was 29% with majority using short term contraceptives. The more educated women, those who had ever used contraceptives and those who desired spacing the children for more than two years had higher odds of using modern contraceptives. These women desired spacing the pregnancy for more than two years but were using short term contraceptive methods. The participants desired to have an average of nine children and considered that the approval of the husband was crucial before use of contraceptives.

### Implications of the study

Understanding the factors that influence uptake of modern contraceptives will help policy makers to engage Somali community leaders on how to make tailored information that is useful in increasing contraceptive awareness among people of Somali decent. This in turn will improve contraceptive uptake for this special group. Further research looking at the reasons underlying the choice of contraceptives and attitudes towards use of long term contraceptives is recommended in this population. Studies investigating ways of increasing male participation in use of modern contraceptives in the Somali community need to be done.

## Data Availability

The datasets used and/or analysed during the current study are available from the corresponding author on request.

## Abbreviations

SOMREC: School Of Medicine Research and Ethics Committee
CPR: Contraceptive prevalence rate
KCCA: Kampala Capital City Authority
UNHCR: United Nations High Commissioner for Refugees
